# A Collaborative Approach to Pain Control Reduces In-hospital Opioid Use and Improves Range of Motion following Total Knee Arthroplasty

**DOI:** 10.7759/cureus.4678

**Published:** 2019-05-16

**Authors:** Christopher Roberts, Devon Foster, Glen G Shi, Elizabeth R Lesser, Michael G Heckman, Joseph L Whalen, Steven R Clendenen, Benjamin K Wilke

**Affiliations:** 1 Orthopedics, Mayo Clinic, Jacksonville, USA; 2 Miscellaneous, Mayo Clinic, Jacksonville, USA; 3 Anesthesiology, Mayo Clinic, Jacksonville, USA

**Keywords:** periarticular injection, total knee arthroplasty, adductor canal block

## Abstract

Introduction: Opioid pain medications are commonly prescribed following orthopedic procedures, with overprescribing of these pain medications implicated as a driver of the current opioid epidemic. In an effort to reduce reliance on opioid pain medications, surgeons are relying on periarticular injections or peripheral nerve blocks. The purpose of this study was to compare numerical rating scale (NRS) pain scores and oral morphine equivalents (OMEs) in patients who underwent primary total knee arthroplasty (TKA) with a periarticular injection alone to those who underwent a collaborative approach with a periarticular injection in the posterior tissue and an adductor canal catheter for anterior knee analgesia.

Methods: In this study, 236 patients underwent a primary TKA between December 2017 and April 2018. Forty patients received an adductor canal catheter and 196 underwent a periarticular injection alone.

Results: We found no difference in patient demographics between the cohorts (p>0.05). The patients that underwent the collaborative approach with a periarticular injection and adductor canal catheter had lower NRS pain scores on post-operative day 0, 1, and 2 (all P≤0.033). These patients demonstrated a reduction of 43% in opioid consumption during the hospitalization (P<0.001). These patients also demonstrated improved range of motion (ROM) (96 vs. 92 degrees) on the day of discharge (P=0.013).

Conclusion: This study provides strong evidence that in patients undergoing TKA, the collaborative approach with the adductor canal catheter and periarticular injection is associated with lower post-operative pain scores, fewer total OMEs per hospital day, and a greater ROM arc prior to discharge compared to patients receiving a periarticular injection alone.

## Introduction

Opioid pain medications are commonly prescribed following orthopedic procedures, with overprescribing of these pain medications implicated as a driver of the opioid epidemic. In one study, it was found that 13% of orthopedic patients became prolonged opioid users (>90 days) following their elective surgery [1]. An additional study demonstrated that orthopedic surgeons provided almost three times the necessary amount of medications to patients following common elective hand surgeries, resulting in a large amount of unused medication [1-3].

In 2005, there were 523,000 total knee arthroplasty (TKA) procedures performed in the United States, with these numbers increasingly yearly [4-7]. This large patient population affords orthopedic surgeons an opportunity to help reduce the opioid burden through advancements in post-operative pain control with less reliance on narcotic medications. One way in which this is accomplished is with increased utilization of periarticular injections and peripheral nerve blocks [8-9]. The adductor canal block (ACB) is a relatively new peripheral nerve block that works on the saphenous nerve in the adductor canal. This has the benefit of controlling anterior knee pain without weakening the quadriceps muscle [10-13]. Previous studies have evaluated pain control with periarticular injections alone compared to peripheral nerve blocks alone [14]. It is less clear how patients do when these blocks are combined in a synergistic approach.

In the current study, our primary aim was to compare post-operative numerical rating scale (NRS) pain scores and oral morphine equivalents (OMEs) used during hospitalization between primary TKA patients who underwent a periarticular injection alone to those who underwent a collaborative approach with the periarticular injection placed by the orthopedic surgeon and directed primarily in the posterior soft tissues and a post-operative adductor canal catheter placed by the anesthesiologist for anterior knee coverage. As a secondary aim, we compared the length of stay (LOS) at the hospital and the range of motion (ROM) arc prior to discharge between the two groups.

## Materials and methods

Study subject

Following the approval of the Institutional Review Board (IRB), a retrospective review was conducted for all 236 patients who underwent unilateral or staged bilateral primary TKAs at our institution between December 2017 and April 2018. These patients were stratified into two cohorts; those who received a periarticular injection alone (196 patients) and those that received a periarticular injection in addition to an adductor canal pain catheter (40 patients) (Ambit; Summit Medical Products, Sandy, Utah). There were six patients who underwent staged bilateral TKAs during the study period. For these patients, only the data from the first hospitalization was included in order to satisfy the statistical assumption of independent measurements.

Periarticular injection and adductor canal pain catheter

The addition of the adductor canal catheter was surgeon dependent. One surgeon included the adductor catheter on all TKA patients. A second surgeon used the catheter only for patients deemed high risk for poor post-operative pain control as determined by their use of narcotics preoperatively. Two additional surgeons did not use the adductor catheter for their patients. All patients that did not receive an adductor canal catheter underwent a periarticular injection for analgesia.

The periarticular injection consisted of 30 mg of ketorolac with weight-based ropivacaine with epinephrine (50-74.9 kg - ropivacaine 200 mg, epinephrine 0.1 mg; 75-99 kg - ropivacaine 300 mg, epinephrine 0.2 mg; 100 kg and greater - ropivacaine 400 mg, epinephrine 0.3 mg). The medication was diluted in normal saline to a final volume of 120 milliliters. This was injected in the soft tissues around the knee prior to closure, focusing on the periosteum and subcutaneous tissue. For patients who only received the periarticular injection, the block was spread evenly through the anterior and posterior soft tissues. If an adductor catheter was to be placed postoperatively, a large portion of the periarticular injection was directed in the posterior soft tissues with limited anterior soft tissue infiltration for improved posterior pain control.

When performed, the catheter (Perifix SoftTip, B. Braun Medical Inc. Bethlehem, Pennsylvania) was placed in the adductor canal below the femoral triangle by the anesthesia team. This catheter was inserted postoperatively in the recovery room while the spinal anesthetic was in effect under ultrasound guidance using a linear probe.

An infusion through the catheter contained ropivacaine 0.2% with a rate of 6 mL/hr, with an hourly patient-controlled on-demand bolus of 6 mL, and was continued for a duration of four days postoperatively. Daily patient rounds were made by the acute pain service to asses functionality of the adductor canal catheter while in the hospital. Patients were discharged home with the catheter and daily phone calls were made to the patient. The catheters were discontinued by a family member on post-operative day four with no reported difficulties or catheter complications.

Data collection and outcomes

Information was collected from chart review regarding baseline patient characteristics (age, gender, body mass index (BMI), pre-operative visual analog pain score, opioid use prior to surgery) and operative information (side of surgery). The pre-operative pain score was calculated based on the preoperative nursing documentation. Opioid use prior to surgery was defined as the use of a narcotic medication within three months prior to the surgical procedure.

Post-operative outcomes were measured and included average, minimum, and maximum NRS pain scores on post-operative days 0, 1, and 2, total OMEs per hospital day, ROM prior to discharge, and LOS. OME data was compiled from a review of the patients’ medication administration record. The NRS pain scores were compiled from nursing documentation, obtained every four hours during the hospital stay per the nursing protocol. These multiple values were then averaged for a single daily value. Although NRS pain scores were measured on post-operative days three and beyond for patients who were still hospitalized, these NRS pain scores were not evaluated as outcomes due to the small number of patients with a LOS longer than two days. The ROM data was extracted from the physical therapy note on the day of discharge. No post-operative falls occurred to any of the patients in the study including patients with adductor canal catheters.

Statistical analysis

The sample median and range were used to summarize continuous variables, while number and percentage were used to summarize the categorical measures. Comparisons of baseline characteristics and operative information between patients who did and did not receive an adductor canal pain catheter were made using a Wilcoxon rank sum test or chi-square test.

Comparisons of outcomes between patients who did and did not receive an adductor canal pain catheter were made using single-variable (i.e., unadjusted) and multivariable regression models that were appropriate for the nature of the given outcome measure. Specifically, linear regression models were used to compare average NRS pain score on post-operative days 0, 1, and 2, total OMEs per hospital day, and ROM arc prior to discharge according to use of an adductor canal pain catheter. Due to their skewed distributions, average NRS pain score on post-operative day 0 and total OMEs per hospital day were examined on the square root scale in linear regression analysis. Regression coefficients and 95% confidence intervals (CIs) were estimated and are interpreted as the difference in the mean outcome measure (on the untransformed or square root scale, as previously described) between patients who did and did not receive an adductor canal pain catheter.

For the ordinal outcome measures of minimum and maximum NRS pain scores on post-operative days 0, 1, and 2 as well as LOS, these were compared according to use of an adductor canal pain catheter using single-variable and multivariable proportional odds logistic regression models. Odds ratios (ORs) and 95% CIs were estimated and are interpreted as the multiplicative increase in the odds of a higher outcome measure for patients who received an adductor canal pain catheter compared to those who did not. The only exception to this was minimum NRS on day 0, which was dichotomized as 0 vs. >0 due to the high concentration of values equal to 0; correspondingly this outcome was analyzed using binary logistic regression models where ORs and 95% CIs were estimated.

All multivariable linear regression, proportional odds logistic regression, and binary logistic regression models were adjusted for age, gender, BMI, pre-operative NRS pain score, narcotic use prior to surgery, and side of surgery. All statistical tests were two-sided and p-values of 0.05 or lower were considered as statistically significant. Statistical analysis was performed using R Statistical Software (version 3.4.2; R Foundation for Statistical Computing, Vienna, Austria).

## Results

For the overall cohort of 236 patients, the median age was 70 years (range: 32 - 98 years). There were 101 males (43%) and 135 females (57%). The median BMI was 30 (range: 18 - 52) and 20% of patients were taking narcotics prior to their operative procedure. Patient baseline characteristics and operative information are summarized in Table 1.

**Table 1:** Baseline characteristics and operative information for the overall series and separately for patients with and without an adductor canal pain catheter BMI: body mass index; NRS: numerical rating scale.

Variable	All patients (N=236)	Adductor canal pain catheter (N=40)	No adductor canal pain catheter (N=196)	P-value
Age (years)	70 (32, 98)	71 (48, 92)	69 (32, 98)	0.25
Gender (male)	101 (42.8%)	17 (42.5%)	84 (42.9%)	0.97
BMI	30 (18, 52)	31 (21, 51)	30 (18, 52)	0.21
NRS pain score	0 (0, 4)	0 (0, 2)	0 (0, 4)	0.063
> 0	65 (27.5%)	15 (37.5%)	50 (25.5%)	0.18
Taking narcotics prior to surgery	47 (19.9%)	8 (20.0%)	39 (19.9%)	0.99
Side of surgery (right)	113 (47.9%)	23 (57.5%)	90 (45.9%)	0.18
The sample median (minimum, maximum) is given for continuous variables. P-values result from a Chi-square test or a Wilcoxon rank sum test.				

We found no statistically significant differences in demographics between patients with and without an adductor canal pain catheter (all P≥0.063). Post-operative outcomes are summarized for the overall patient series in Table 2.

**Table 2:** Post-operative outcomes in the overall patient series NRS: numerical rating scale; OME: oral morphine equivalent; ROM: range of motion.

Outcome	All patients (N=236)
Average NRS	
Day 0	1.4 (0.0, 7.8)
Day 1	3.7 (0.0, 9.0)
Day 2	3.6 (0.0, 9.0)
NRS Minimum	
Day 0	0 (0, 5)
Day 1	1 (0, 8)
Day 2	2 (0, 9)
NRS Maximum	
Day 0	5 (0, 10)
Day 1	6 (0, 10)
Day 2	6 (0, 10)
Total OMEs per hospital day	68 (0, 352)
ROM arc prior to discharge (degrees)	93 (32, 125)
Length of stay (days)	
1	68 (28.8%)
2	132 (55.9%)
3	33 (14.0%)
4-6	3 (1.3%)
The sample median (minimum, maximum) is given for continuous variables	

Post-operative outcomes are compared between patients with and without an adductor canal pain catheter in Table 3.

**Table 3:** Comparisons of post-operative outcomes between patients who did and did not receive an adductor canal pain catheter NRS: numerical rating scale; OME: oral morphine equivalent; ROM: range of motion; CI: confidence interval.

	Median (Minimum, Maximum)			Single-variable analysis		Multivariable analysis	
Outcome measure	No adductor canal pain catheter (N=196)	Adductor canal pain catheter (N=40)	Association measure	Estimate (95% CI)	P-value	Estimate (95% CI)	P-value
Average NRS pain score							
Day 0	1.4 (0.0, 7.1)	0.9 (0.0, 7.8)	Regression coefficient	-0.25 (-0.48, -0.02)	0.033	-0.33 (-0.54, -0.12)	0.002
Day 1	3.9 (0.0, 9.0)	2.5 (0.0, 7.4)	Regression coefficient	-1.00 (-1.67, -0.34)	0.003	-1.06 (-1.66, -0.45)	0.001
Day 2	3.7 (0.0, 9.0)	2.5 (0.0, 6.0)	Regression coefficient	-1.20 (-1.96, -0.41)	0.003	-1.46 (-2.21, -0.70)	<0.001
Minimum NRS pain score							
Day 0	0 (0, 5)	0 (0, 3)	Odds ratio	0.66 (0.19, 3.06)	0.55	1.80 (0.32, 14.82)	0.54
Day 1	1 (0, 8)	0 (0, 4)	Odds ratio	0.40 (0.20, 0.80)	0.010	0.32 (0.15, 0.70)	0.004
Day 2	2 (0, 9)	0 (0, 4)	Odds ratio	0.51 (0.23, 1.12)	0.090	0.46 (0.19, 1.08)	0.073
Maximum NRS pain score							
Day 0	5 (0, 10)	3 (0, 10)	Odds ratio	0.41 (0.22, 0.75)	0.004	0.27 (0.14, 0.51)	<0.001
Day 1	6 (0, 10)	5 (0, 10)	Odds ratio	0.52 (0.29, 0.95)	0.030	0.40 (0.21, 0.74)	0.004
Day 2	6 (0, 10)	5 (0, 10)	Odds ratio	0.49 (0.24, 1.02)	0.057	0.27 (0.12, 0.60)	0.001
ROM arc prior to discharge	92 (32, 125)	96 (70, 115)	Regression coefficient	4.48 (0.14, 8.83)	0.043	5.44 (1.16, 9.72)	0.013
Total OMEs per hospital day	76 (0, 344)	43 (0, 173)	Regression coefficient	-2.16 (-3.12, -1.20)	<0.001	-2.13 (-2.95, -1.31)	<0.001
Length of stay	2 (1, 6)	2 (1, 3)	Odds ratio	1.20 (0.62, 2.33)	0.59	1.03 (0.52, 2.06)	0.93
Regression coefficients, 95% CIs, and p-values result from linear regression models. Odds ratios, 95% CIs, and p-values result from proportional odds logistic regression models for all outcome measures except minimum NRS pain score on day 0, where binary logistic regression was used due to the small number of patients with non-zero values. Multivariable models were adjusted for age, gender, BMI, pre-operative NRS pain score, narcotic use prior to surgery, and side of surgery. Regression coefficients are interpreted as the difference in the mean outcome measure (on the square root scale for average NRS pain score on day 0 and total OMEs per hospital day) when comparing patients who received an adductor canal pain catheter to patients who did not receive an adductor canal pain catheter (i.e. the reference group). Odds ratios are interpreted as the multiplicative increase on the odds of a greater outcome measure for patients who received an adductor canal pain catheter to patients who did not receive an adductor canal pain catheter (i.e. the reference group) with the exception of the minimum NRS pain score on day 0 outcome measure. For that specific outcome measure, odds ratios are interpreted as the multiplicative increase on the odds of minimum NRS pain score greater than 0 for patients who received an adductor canal pain catheter to patients who did not receive an adductor canal pain catheter (i.e., the reference group).							

**Figure 1:**
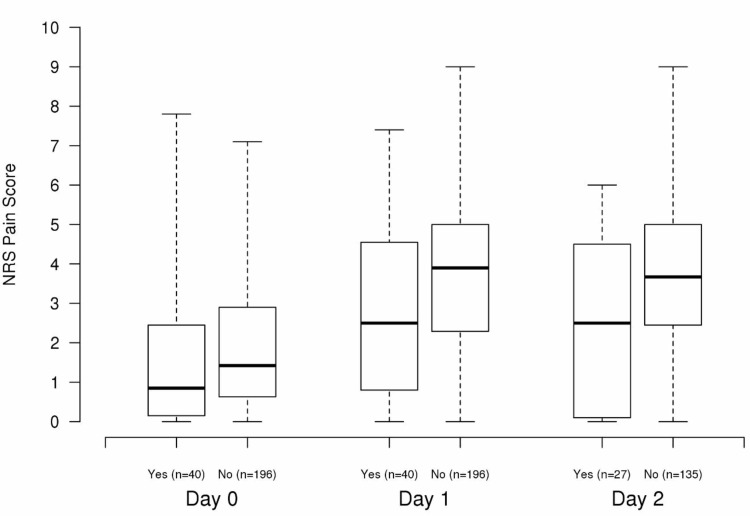
Boxplots of average numerical rating scale (NRS) pain score on post-operative days 0, 1, and 2 for patients with an adductor canal pain catheter (denoted as “yes”) and patients without an adductor canal pain catheter (denoted as “no”)

In comparison to patients who did not receive an adductor canal pain catheter, those patients who did receive a pain catheter had significantly lower NRS pain scores on post-operative days 0, 1, and 2 in both single-variable analysis and multivariable analysis adjusting for age, gender, BMI, pre-operative NRS pain score, narcotic use prior to surgery, and side of surgery (all P≤0.033) (Figure 1).

**Figure 2:**
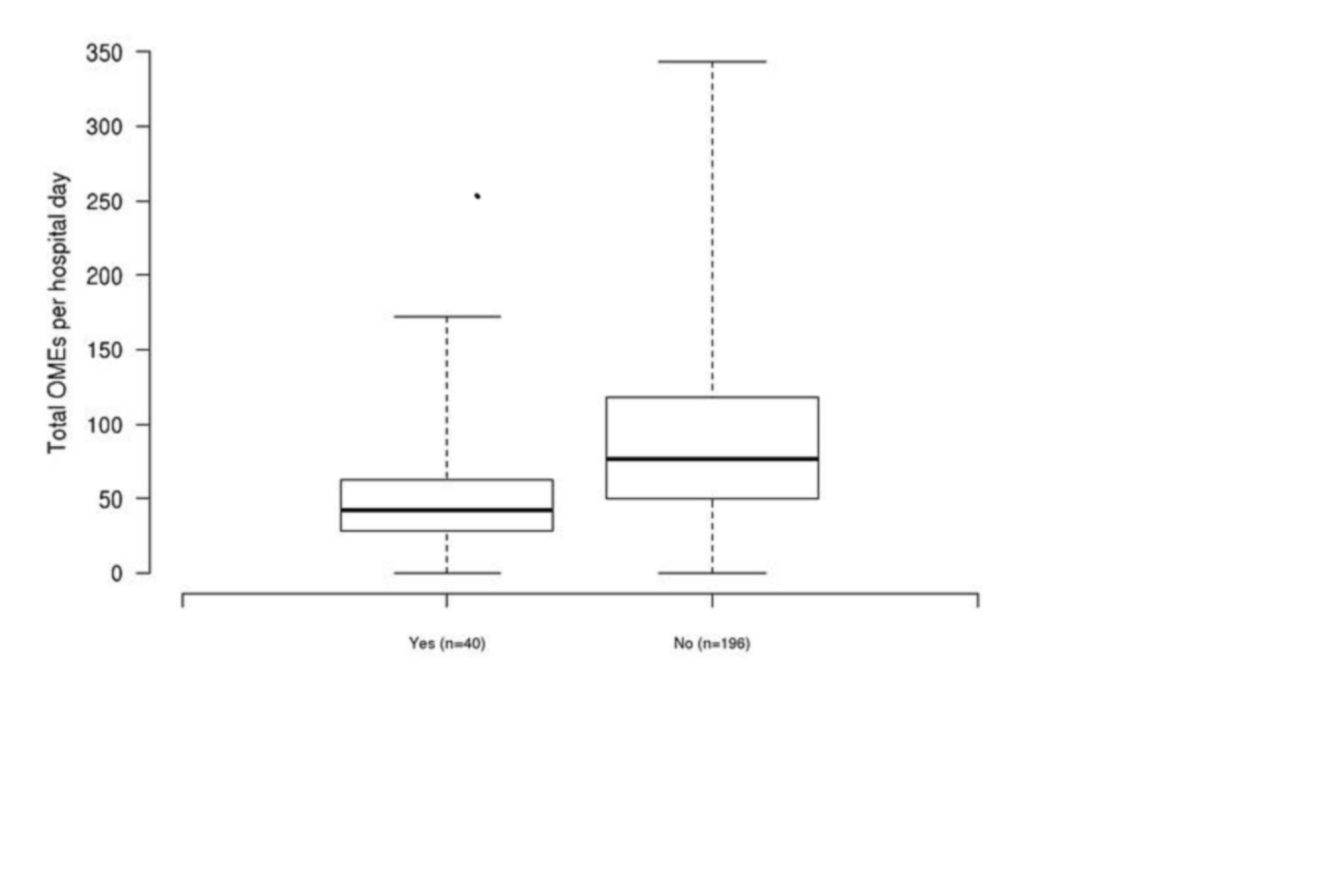
Boxplots of total oral morphine equivalents (OMEs) per hospital day for patients with an adductor canal pain catheter (denoted as “yes”) and patients without an adductor canal pain catheter (denoted as “no”)

Additionally, the pain catheter cohort had significantly lower total OMEs used per hospital day compared to the periarticular cohort (median: 43 vs. 76) in single-variable and multivariable analysis (both P<0.001) (Figure 2).

**Figure 3:**
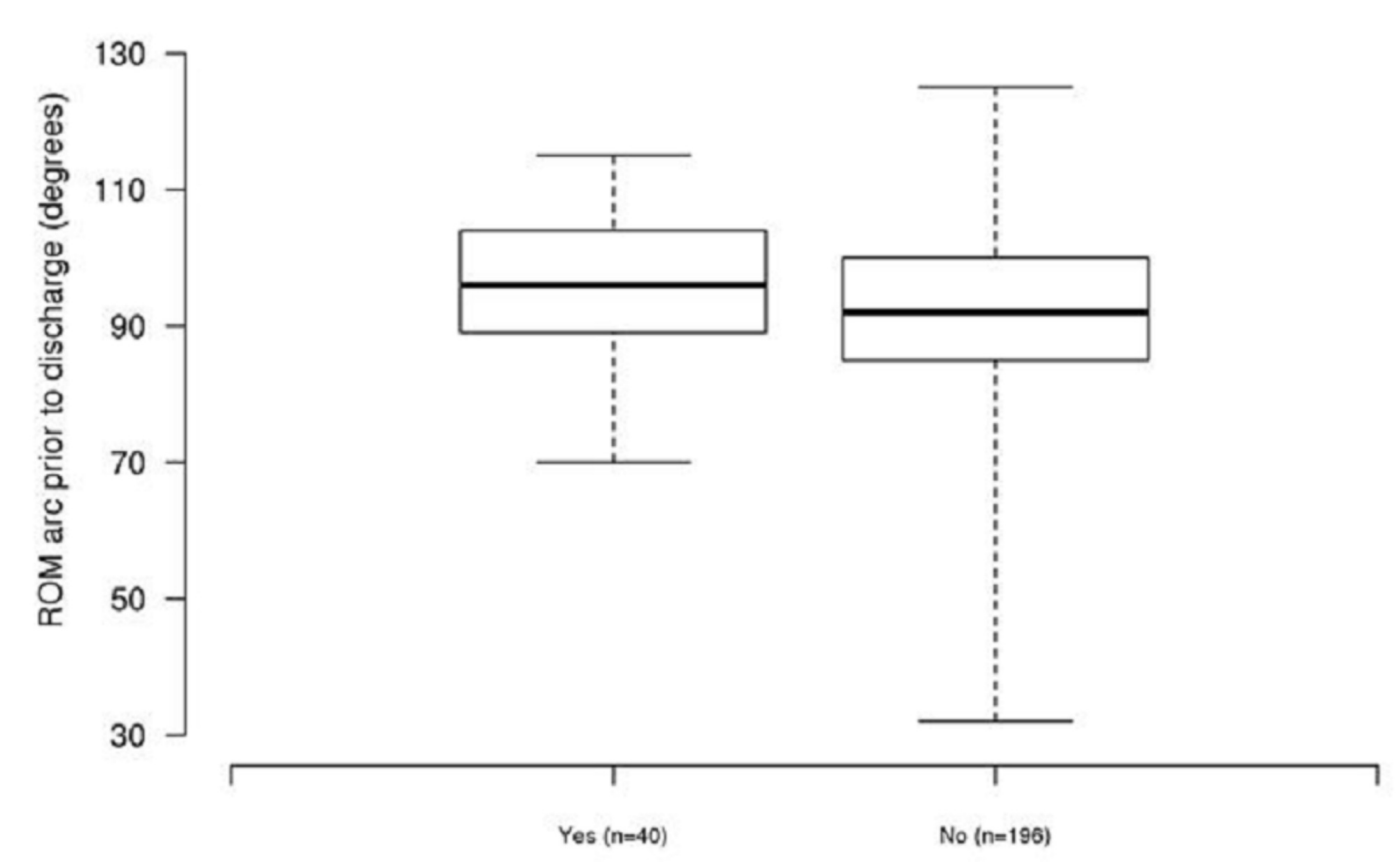
Boxplots of range of motion (ROM) arc prior to discharge for patients with an adductor canal pain catheter (denoted as “yes”) and patients without an adductor canal pain catheter (denoted as “no”)

The adductor canal catheter group also demonstrated a significantly higher ROM arc (median: 96 degrees vs. 92 degrees) prior to discharge in both single-variable analysis (P=0.043) and multivariable analysis (P=0.013) (Figure 3).

In addition, minimum NRS pain score on post-operative day 0 and maximum NRS pain score on post-operative days 0, 1, and 2 were all significantly lower in multivariable analysis for patients who received an adductor canal pain catheter (all P≤0.004). There was no significant difference in post-operative LOS between the two treatment groups in single-variable analysis (P=0.59) or multivariable analysis (P=0.93).

## Discussion

Post-operative pain can be difficult to control and the lack of relief can lead to significant distress to patients, decreased rehabilitation participation, increased the LOS in the hospital, and increased hospital costs [15-19]. Opioid medications have been a mainstay of pain management strategies following orthopedic surgery; however, due to the ongoing opioid epidemic, new strategies are needed to help curb the reliance on these medications.

The ACB has gained recent popularity as an effective alternative to periarticular injections alone, with several studies showing equivalent pain relief [11,13]. The adductor catheter is directed at the saphenous nerve, which predominately innervates the anterior knee, leaving the posterior knee uncovered. It is unknown if the addition of a periarticular injection, predominately focusing on the posterior soft tissues will provide a synergistic effect to the adductor block to improve pain control compared to the periarticular injection alone.

In the current investigation, we observed significantly lower average NRS pain scores in the adductor canal catheter group at post-operative day 0, day 1, and day 2 compared to the periarticular injection alone. While statistically significant, the decrease in one point on the NRS scale is likely not clinically significant. However, combined with the improved pain score was a 43% reduction in mean daily OMEs in the catheter group compared to the periarticular group. This demonstrates a substantial reduction in narcotics required to alleviate pain postoperatively.

In addition to improved pain control with fewer narcotics, the adductor canal catheter group also demonstrated an improved ROM arc compared to the periarticular group on the day of discharge. Specifically, the multivariable analysis indicated that mean ROM arc was greater than 5 degrees higher for the adductor canal catheter patients. This data is supported by previous research that demonstrated improved quadriceps muscle strength, better ambulation ability, and faster functional recovery with an adductor canal catheter for TKA patients [12-13]. Similarly, a study by Perlas et al. demonstrated improved early ambulation with a single-shot ACB and a periarticular block (PAB) compared to the periarticular group alone [10]. A study by Kampitak compared single shot ACB and PAB with ACB and placebo. The ACB and PAB showed a six-hour delay in rescue analgesia. In our study, we were able to show prolonged analgesia with a catheter with no complications of home catheters. Future studies will be needed to determine if the improved ROM continues beyond the acute hospitalization.

Several limitations of this study are important to note. First, this was a retrospective review and suffers from limitations inherent with that type of study design. Second, these results were from a single center with anesthesiologists trained in the placement of the adductor canal catheter and therefore the results may not be reproducible at all centers. Finally, the sample size of the adductor pain catheter group was relatively limited. However, we were able to demonstrate a number of statistically significant differences in post-operative outcomes between patients who did and did not receive an adductor canal pain catheter despite the relatively small sample size of the former group.

## Conclusions

The results of this study provide strong evidence that in patients undergoing TKA, receiving an adductor canal catheter with a periarticular injection directed primarily at the posterior soft tissue structures is associated with lower post-operative NRS pain scores, fewer total OMEs per hospital day, and a greater ROM arc prior to discharge compared to patients receiving a periarticular injection alone. This suggests that a collaborative approach between the orthopedic surgeon and anesthesiologist may provide the optimal strategy for post-operative pain control. Future studies are needed to determine if these benefits continue to be observed following hospital discharge.
